# Novel calreticulin-nanoparticle in combination with focused ultrasound induces immunogenic cell death in melanoma to enhance antitumor immunity

**DOI:** 10.7150/thno.42243

**Published:** 2020-02-10

**Authors:** Sri Nandhini Sethuraman, Mohit Pratap Singh, Girish Patil, Shitao Li, Steven Fiering, P. Jack Hoopes, Chandan Guha, Jerry Malayer, Ashish Ranjan

**Affiliations:** 1 Center for Veterinary Health Sciences, Oklahoma State University, Stillwater, Oklahoma 74074; 2 Geisel School of Medicine, Dartmouth, Hanover, NH03755; 3 Albert Einstein College of Medicine, Bronx, New York 10461

**Keywords:** Immunogenic cell death, calreticulin, nanoparticle, melanoma, focused ultrasound

## Abstract

**Rationale**: Some studies have shown that the local activation of immunogenic cell death (ICD) by upregulating calreticulin (CRT) expression in solid tumors can improve antitumor effects. Although a promising approach, a key current challenge in ICD tumor therapy is the absence of a clinically translatable method for reproducibly inducing the CRT expression. Herein, we report a novel calreticulin-nanoparticle (CRT-NP) that enhances ICD and synergizes with focused ultrasound (FUS) to achieve local and systemic antitumor effects.

**Methods**: Full-length clone DNA of calreticulin was encapsulated in NPs made from DOTAP and cholesterol. Three CRT-NP intratumoral injections of 20 µg each were given 2 days apart, and FUS heating (42-45°C, ~15min) was applied sequentially 24h after each injection to induce ICD. To investigate ICD specific immune effect, the splenocytes of mice vaccinated with CRT-NP (± FUS) treated B16F10 cells were evaluated ex-vivo for TRP-2 antigen specific immunity. Additionally, the long-term protection was evaluated by re-challenging with the melanoma cells in the flank regions of tumor bearing mice.

**Results**: CRT-NP plus FUS (CFUS) upregulated CRT expression, expanded the population of melanoma TRP-2 specific functional CD4+ and CD8+ T cells and tumor-suppressing M1 phenotype, and increased PD-1 and PD-L1 marker expression in the T cells. Therapeutically, CFUS suppressed B16 melanoma growth by >85% *vs*. that seen in untreated controls, and >~50% *vs*. CRT-NP or FUS alone, and prevented tumor growth in distal untreated sites.

**Conclusions**: CRT-NP amplifies the FUS and ICD therapeutic outcomes against melanoma, suggesting that the proposed combinatorial methodology may be clinically translatable.

## Introduction

The ability of the immune system to recognize, attack and eliminate cancerous cell is well established 1-5. However, several tumor types (e.g. melanoma, pancreatic, breast) can manifest a variety of immunosuppressive mechanisms to evade immune attack 6-8. Reversing the protective immune suppression and stimulating immune cell activation is the key goal of cancer immunotherapy 9, 10. Some recent studies have shown that chemo- and radiotherapy can induce the release of damage associated molecular patterns (DAMPs) such as calreticulin (CRT), ERP57, HMGB1, ATP, and heat shock proteins from dying cells 11-15. This phenomenon is called an immunogenic cell death (ICD) and, when tumor cells experience ICD, the immune stimulatory effects of ICD improve the populations of tumor antigen presenting and cytotoxic T-cell (CTL) cells, enhancing antitumor immune responses 11, 13, 16-20. In particular, CRT proteins enhance the phagocytosis and immunogenic recognition of dying cancer cells by antigen presenting cells (APCs)^12^, ^14^, ^21^ and improve interactions with tumor infiltrating leukocytes by modulation ICAM-1 and VCAM-1 on tumor endothelial cells 14. Additionally, tumor-associated macrophages expressing activated CRT on cell surfaces exhibit efficient phagocytosis of cancerous cells 22. Although able to contribute to the needed immune stimulation, CRT productions are often inconsistent in most solid tumors and by themselves generally are not sufficient to generate an effective antitumor immune response 23. Thus, there is a critical need to develop novel approaches that improve local CRT-based ICD outcomes in immunosuppressive tumors.

The objective of this study was to develop novel calreticulin nanoparticle (CRT-NP) and combine it with focused ultrasound (FUS) for ICD based immunomodulation in melanoma. Our CRT-NP is a biocompatible liposome-based gene delivery agent. Unlike viral vectors that risk mutagenic integration with host cells, the liposomes are a promising yet safer gene-drug delivery technology 18. FUS is an extracorpeal treatment device that delivers focused sound waves non-invasively, providing a powerful tool for clinical administration of anatomically-specified thermal effect in soft tissues 24, 25. We and others have shown that FUS-induced thermal effect modifies the tumor microenvironment to impart several benefits including enhanced response to chemotherapy, tumor antigen release, expression of heat-shock proteins, upregulation of pro-phagocytic signals such as CRT, and overall tumor immunity stimulation compared to conventional treatment 24, 26-29. Based on this premise, we hypothesized that the direct intratumoral (in-situ) injection of CRT-NP in easily accessible melanoma tumors and combination with FUS heating (42-45ºC) would abrogate the aberrant and tumor-suppressive factors and trigger the clearance of melanoma cells.

To test our hypothesis, we characterized the infiltration of activated macrophages and CD8+ T cell, and the expression of innate (CD47) and adaptive markers (PD-1, PD-L1, etc.) in the immunosuppressive B16F10 model. CD47 membrane protein interacts with receptor signal regulatory protein on immune cells to inhibit phagocytosis by the macrophages 30, 31. Like CD47, the expression of checkpoints namely PD-1 on T cells impair the interaction of effector T cells and macrophages with tumor cells via the PD-1/PD-L1 axis 32, 33. Our data suggest that FUS and CRT-NP combination transforms the melanoma tumor immune microenvironmental factors, aiding immune clearance. Thus, the proposed combinatorial modality has the potential to ease the clinical translation of ICD approaches in clinics.

## Material and Methods

### Materials

1,2-dioleoyl-3-trimethylammonium-propane (DOTAP) was purchased from Corden Pharma (Wouburn, MA, USA) or Avanti polar lipids (Alabaster, Alabama, USA) and Cholesterol from Calbiochem (San Diego, CA, USA). B16F10 murine melanoma cells were from Dr. Mary Jo Turk at Geisel School of Medicine at Dartmouth (Hanover, NH). B16F10 cells were cultured in DMEM supplemented with 10% fetal bovine serum (FBS) and 1% streptomycin/penicillin. Plasmids containing CRT genes controlled by the CMV promoter were obtained from Sino Biological (Collegeville, PA, USA). Fluorochrome-conjugated monoclonal antibodies (mAbs) for flow cytometry were purchased from BioLegend (San Diego, CA, USA) and are listed here: APC anti-CD4 (GK1.5), PE anti-CD3 (145-2C11), PERCP anti-F4/80 (BM8), APC-Cy7 anti-MHCII (M5/114.15.2), APC-CY7 anti-IFN-γ (XMG1.2), APC anti-CD11c (N418), FITC anti-CD45.2 (104), FITC anti-CD86 (GL-1), FITC anti-Granzyme B (GB11), FITC anti-PD-1 (29F.1A1.2), Alexa fluor 700 anti-CD206 (C068C2), PE or APC anti-CD11b (M1/70), FITC anti-Gr-1 (RB6-8C5), Pacific blue anti-ICAM-1 (YN1/1.7.4), PE anti-PD-L1 (10F.9G2), and PE anti-CD47 (miap301). FITC anti-CD8a (53-6.7) and PE anti-Foxp3 (R16-715) were purchased from BD Biosciences (San Jose, CA, USA). Alexa fluor 647 anti-CRT (EPR3924) was obtained from Abcam (Cambridge, MA, USA).

### Synthesis and characterization of CRT-NP

Full-length clone DNA of human CRT cloned into pCMV3 vector was used (HG13539-ACR, Sino Biological Inc., Wayne, PA, USA). For CRT-NP synthesis, a lipid film was hydrated in 10 mM HEPES buffer (pH 7.4) at 55°C, and the lipid suspension was then extruded five times through filters of 200 nm pore size to yield homogeneous liposomes 34. Next, a one-step method for loading the plasmid was developed by adding the pDNA solution in the liposomes vial (1:10, wt/wt), gently mixing them by pipetting, and incubating at room temperature for 30 min. The resultant CRT-NPs were characterized for plasmid encapsulation by the gel retardation assay in 1% agarose pre-cast gels containing ethidium bromide. A control sample of free pCRT as well as blank NPs were loaded onto the gels and the gels were run at 80 V on a Bio-Rad electrophoresis system. DNA dose was 0.2 µg per lane. Approximately 60 minutes after beginning the run, the gels were observed for plasmid migration. The CRT-NPs were also characterized in physiological buffers at room temperature by size (z-average) and zeta-potential using dynamic light scattering (DLS) with a Brookhaven ZetaPALS instrument (Holtsville, NY, USA). Furthermore, transmission electron microscopy (TEM) as previously published was performed to assess the morphology of CRT-NP using the JEOL JEM-2100 TEM (JEOL USA, Peabody, MA, USA) 26.

### Assessment of transfection and uptake efficiency of CRT-NPs in melanoma cells in vitro with fluorescence microscopy and flow cytometry

For in vitro transfection assessment, full-length clone DNA of human CRT with a C terminal OFP Spark tag cloned into pCMV3 vector was used. 1 x 10^5^ B16F10 cells per well were seeded in 24-well plates for 18-24h prior to transfection. On the day of transfection, cell culture medium was replaced with serum free medium and the cells were incubated with CRT-NPs (2 µg total DNA per well) at 37°C and 5% CO_2_ for 5h. Next, the cells were rinsed to remove non-phagocytosed NPs and incubated for an additional 48h with DMEM containing 10% serum to assess the CRT expression. To compare the transfection efficiency, CRT plasmid was complexed with commercially available Lipofectamine^TM^2000 (LF2000) and used as a positive control according to manufacturer's instructions. Fluorescence imaging of CRT was performed with the RFP filter cube (ex/em of 531/593 nm) using Biotek Cytation 5 cell imaging multimode reader (Winooski, VT, USA) and the images were acquired using Gen5 Image+ software version 3.08.01. Image acquisition and display parameters were kept constant to allow for qualitative comparison. Transfected cells (impermeabilized) were stained with surface targeting Alexa fluor 647 anti-CRT antibody and cell surface expression of CRT was quantified by flow cytometry in an LSRII analyzer (BD Biosciences, Franklin Lakes, NJ, and U.S.A.). In addition, we characterized the CRT-NP uptake efficiencies in B16F10 cells using flow cytometry. Briefly, B16F10 cells were incubated with coumarin dye labeled CRT-NPs for 5, 8, and 24h, and the mean fluorescence intensity (MFI) was assessed and compared (n=3).

### FUS treatment methodology for in vitro and in vivo assays

An imaging and therapeutic ultrasound system (Alpinion medical systems, Bothell, WA, USA) was used for all FUS exposures. The system consists of FUS transducer with a 1.5 MHz central frequency, 45 mm radius, and 64 mm aperture diameter with a central opening of 40 mm in diameter and an automated motion stage to achieve accurate positioning perpendicular to FUS beam axis. FUS treatment parameters used were as follows: 5 Hz frequency, 50% duty cycle, and 6 W power (equivalent to 3.5 W acoustic power). This method achieved a mean target temperature of 42-45°C at the focus inside the tumor (measured by inserting a fiber optic temperature sensor; Qualitrol, Quebec, Canada). The duration of FUS exposure at the focus was 15 min. For tumor treatments, the center of the tumor was aligned at a fixed focal depth for efficient coverage voxel size (5 x 5 x 12 mm) using a sector vortexed lens 24. As the tumor grew, the focal point was rastered to cover the entire tumor. For in-vitro FUS treatments, the transducer was used without lens to achieve a coverage of 1 x 1 x 10 mm. An integrated VIFU-2000 software was used to define target boundary and slice distance in x, y, and z directions for automatic rastering of the transducer for 15 min.

### Assessment of CRT and CD47 expression in B16F10 cell membranes post treatments, and evaluation of tumor growth in vivo

5 x 10^5^ B16F10 cells per well were seeded in 6-well plates 18-24h prior to transfection. On the day of transfection, the cells were incubated with CRT-NPs (1 µg DNA per well) at 37°C and 5% CO_2_ for 48 h. Cells were then harvested and re-suspended in sterile PBS and transferred into 0.5 ml thin-walled PCR tube. The tube was placed vertically with its conical bottom aligned within the beam focus of the FUS transducer for 15 min as described previously 26, 35. Temperature elevation (~42-45°C) in the cell suspension with FUS was monitored using a fiber optic temperature sensor. Following FUS, the treated cells were incubated for an additional 24 h at 37°C and 5% CO_2_. Non-transfected cells were used for control. Finally, the surface expression of CRT and CD47 (n=3) was determined using flow cytometry. Stained cells were run in an LSRII analyzer (BD Biosciences, Franklin Lakes, NJ, and U.S.A.) and datasets were analyzed using FlowJo software v.10.2 (Treestar Inc, Ashland, OR, USA). Compensations were performed with single-stained UltraComp eBeads or cells. For all channels, positive and negative cells were gated on the basis of fluorescence minus one control. In addition, B16F10 cells transfected with CRT-NPs (followed by ± FUS) were gently rinsed, scraped, and re-suspended in sterile PBS. 4 x 10^6^ CRT-NPs and CFUS treated B16F10 cells (~50% viable assessed by trypan blue) were inoculated s.c. in the flank region of each mouse as a vaccine (n=5 mice/group). Tumor growth was monitored for 4 weeks. After 4 weeks, mice were sacrificed.

### Mouse melanoma CRT-NP and FUS administration, and assessment methodology in primary and re-challenge therapeutic studies

All animal-related procedures were approved and carried out under the guidelines of the Oklahoma State University Animal Care and Use Committee. B16F10 cells in DMEM supplemented with 10% v/v fetal bovine serum (FBS) and 1% v/v streptomycin/penicillin at 80- 90% confluency were harvested, washed, and diluted with sterile cold PBS to generate a dose of 0.5 × 10^6^ cells in 50 μL per mouse. Tumor cells were injected in the flank region using a 27-gauge needle (BD, Franklin Lakes, NJ, USA). Mice tumor volume was measured daily by serial caliper measurements (General Tools Fraction™, New York, NY, USA) using the formula (length × width^2^)/2, where length was the largest dimension and width was the smallest dimension perpendicular to the length. Treatments were initiated when tumors reached a volume of 40-60 mm^3^ (n=4-7). We compared the following groups: 1) Control, 2) FUS, 3) CRT-NP, and 4) CFUS. Three CRT-NP intratumoral injections (20 µg DNA per injection) were given 2 days apart. FUS hyperthermia (~42-45°C) was applied 24 h after each CRT-NP injection. FUS or CRT-NPs alone cohorts received three treatments on alternating days. Untreated tumor-bearing mice served as controls for evaluation of immune changes and abscopal effects. For tumor re-challenge, tumor bearing mice (n=4-5) were injected with 1 × 10^5^ cells/50 µL s.c. on the contralateral flank 7 days post-treatment of primary tumors as previously reported 36, 37. The differences between groups in resistance to rechallenge at distant site were assessed.

### Evaluation of ICD mediated immune effects in treated tumors, lymph node and spleen tissues using flow cytometry

For in vivo studies, mice were sacrificed 26-28 days post inoculation, and the tumors (primary), tumor draining lymph nodes (dLNs) and the spleen were excised, weighed, and processed for flow cytometry, western blot, and immunofluorescence. For flow cytometry, harvested tissues were used on the same day. Single-cell suspensions obtained from mechanical disruption of the tumors followed by enzymatic digestion (200 U/mL collagenase IV; Life Technologies, NY, USA) were filtered through a 70 μm cell strainer (Corning Inc, Corning, NY). Cells were stained with combinations of the indicated fluorochrome-conjugated anti-mouse antibodies for 30 min in the dark on ice. Antibody combinations used to distinguish immune cell populations were as follows: CD45+ (Tumor infiltrating leukocytes; TILs), CD3+, CD4+ (CD4+ T or helper Th cells), CD3+, CD8+ (CD8+ T cells), CD11b+, F4/80+ (macrophages), CD11b+, F4/80+, MHCIIhi (M1 macrophages) and CD86+ (activated M1 macrophages), CD11b+, F4/80+ MHCII lo/neg, CD206+ (M2 macrophages), CD11b+ CD11c+, F4/80-, MHCII+ (dendritic cells), CD11b+ Gr-1+ (Myeloid-derived suppressor cells, MDSCs), PD-L1+ TILs and tumor cells, and PD-1+ CD3+ CD8+ T cells. For detecting IFN-γ, Granzyme-B, and Foxp3 positive T cells, cells were washed after surface marker staining, fixed and permeabilized with transcription factor buffer set (BD Biosciences, Franklin Lakes, NJ, U.S.A.) and incubated with APC Cy7 anti-IFN-γ, FITC anti-Granzyme-B or PE anti-Foxp3 antibody for 30 min in the dark on ice. Stained cells were run in an LSRII analyzer (BD Biosciences, Franklin Lakes, NJ, and U.S.A.) within 24h. Compensations were performed with single-stained UltraComp eBeads or cells. Datasets were analyzed using FlowJo software v.10.2 (Treestar Inc, Ashland, OR, USA). For all channels, positive and negative cells were gated on the basis of fluorescence minus one control.

### Establishment of melanoma specific immunity of CRT-NP and CFUS treatments

To determine melanoma specific immunity, spleen (n=3-4) and dLNs (n=3) from the surviving mice were stimulated ex-vivo with melanoma specific differentiation antigen tyrosinase-related protein 2 (TRP-2) peptide for 8h to evaluate generation of TRP-2 melanoma antigen specific immunity 36, 38. Briefly, 1-2x10^6^ splenocytes and dLN cells were incubated with 5 µg/ml TRP-2 peptide for 8 h in the presence of Brefeldin A (eBioscience, 1000X solution) at 37°C and 5% CO_2_. Treated cells were washed with PBS and stained with CD45, CD3, CD4, CD8, and IFN-γ antibodies for flow cytometry. For immunofluorescence and western blot estimation, tissues were snap-frozen in liquid nitrogen and stored at -80°C until further analysis.

### Assessment of PD-L1 expression in tumor lysates by western blotting

For the assessment of PD-L1 expression (n=4-7), crude membranes were extracted using Mem-PER™ Plus Membrane Protein Extraction Kit (ThermoFisher Scientific) according to the manufacturer's instructions. Equal amounts of protein (10 μg) were resolved in 4-20% Mini-PROTEAN polyacrylamide gel (BioRad, CA, USA) and were subsequently transferred onto a nitrocellulose membrane using a BioRad Turbo Trans system. After blocking (5% non-fat dry milk), membranes were incubated at 4°C overnight with anti-mouse PD-L1 (1:1000, Sino Biological, 50010-732). anti-mouse GAPDH (1:10000, Invitrogen, AM4300) and anti-Na+/K+ ATPase (1:3000, Abcam, ab7671 and 1:1000, Cell Signalling Technology, 3010). This was followed by incubation with secondary antibodies conjugated to horseradish peroxidase (1:10000, rabbit or goat anti-mouse, Jackson ImmunoResearch Inc., PA, USA) at room temperature for 1h. The blots were developed using the ECL kit (Thermo Scientific, Rockford, IL) and imaged by Amersham imager 600 system (GE Healthcare Bio-Sciences, Uppsala, Sweden). Na+/K+ ATPase was used as a loading control. Densitometric analyses were performed using ImageJ 1.51 software (NIH), and the data were normalized relative to appropriate GADPH controls.

### Immunofluorescence staining of tumor sections for CRT expression assessment

Tumor sections of 5 μm thickness embedded in OCT were permeabilized with acetone for 5 min and incubated with 1% BSA in phosphate-buffered saline for 2h to block non-specific protein-protein interactions. Tissue sections were incubated overnight at 4°C with primary anti-rabbit anti-calreticulin antibody (Pierce, PA5-25922) according to the manufacturer's recommendations. This was followed by incubation with secondary antibody conjugated to Alexa Fluor Plus 647 (Thermo Scientific, A32733) at room temperature for 1h. Fluorescently-labeled tissues were mounted with medium containing DAPI for cell nuclei visualization (Vector Laboratories). Cell nuclei were visualized at an exposure time of 5 ms (ex/em of 365/440), and CRT was imaged at an exposure 10 ms (ex/em of 650/672). Image acquisition and display parameters were constant for different groups to allow for qualitative comparison.

### Analysis of IL1-β and TNF-α in tumor samples by ELISA

50 μl of the tumor supernatant from homogenized tumors samples were utilized for IL1-β (n=3) and TNF-α (n=7-8) using Quantikine ELISA kit (R&D Inc., MN, USA) according to the manufacturer's instructions.

### Statistical analyses

Statistical analyses were performed using GraphPad Prism 8.0 software (GraphPad Software Inc, La Jolla, CA, USA). Data are presented as mean ± SEM unless otherwise indicated. For analysis of 3 or more groups, a one-way ANOVA test was performed followed by Fisher's LSD without multiple comparisons correction. The overall *P* value for Kaplan-Meier analysis was calculated by the log-rank test. Analysis of differences between 2 normally distributed test groups was performed using an unpaired t-test assuming unequal variance. Correlations between PD-1+ CD8+ T cells and granzyme B+ CD8+ T cells were analyzed using a Pearson correlation test, pooling data across the different treatment groups. P values less than 0.05 were considered significant.

## Results

### CRT-NPs efficiently encapsulated the plasmid and induced intracellular CRT expression, and synergized with FUS in vitro and in vivo by modulating CD47 to CRT ratio

**Figure 1:**
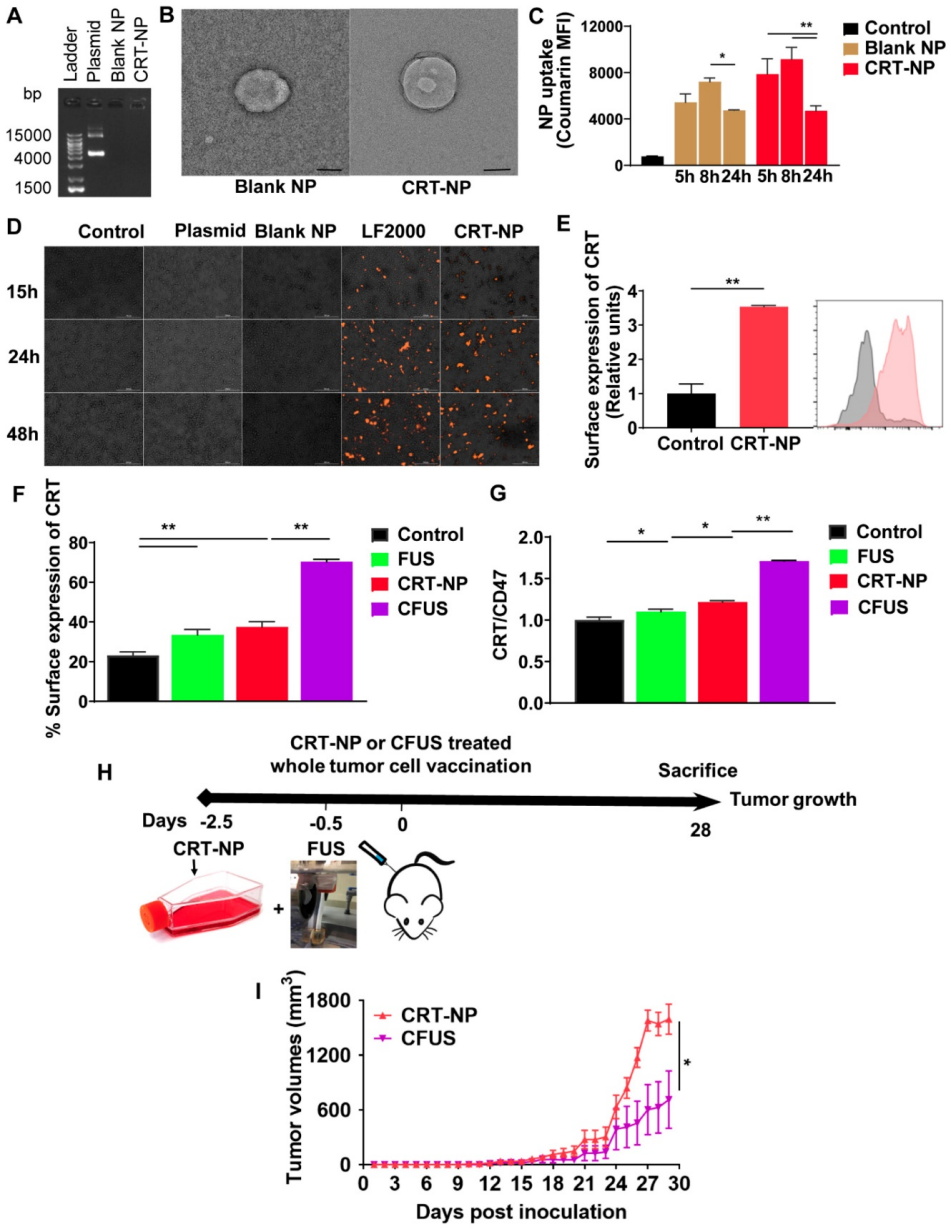
Combination of FUS with CRT-NPs therapy increased the CRT expression and CRT/CD47 ratio. (A) Characterization of CRT-NP using gel retardation assay suggested complete encapsulation of CRT plasmid in the NPs. In contrast, blank NPs and CRT-NPs demonstrated no band. (B) Transmission electron microscopy of CRT-NP demonstrated a typical core-shell morphology with the encapsulated plasmid compared to blank NP Scale bar is 100 nm. (C) Quantification of coumarin labeled CRT-NP uptake using flow cytometry showed efficient uptake from 5-8h similar to blank NPs. The median fluorescence intensity (MFI) of coumarin reduced at 24h likely due to NP lysis over time. (D) Fluorescence imaging of B16F10 cells incubated with CRT-NPs (2 µg DNA) showed efficient transfection and protein expression (orange) similar to Lipofectamine^TM^2000 (LF2000). (E) Flow cytometric analysis of surface expression of CRT 48 h after CRT-NP transfection (4 µg DNA) is shown in bar graph and histogram plot (n=3). Control (grey peak) indicates non-transfected cells. (F and G) Flow cytometric analysis of surface CRT expression (F) and CRT to CD47 ratio (G) in B16F10 cells transfected with CRT-NP (1 µg DNA) for 40-42h followed by FUS treatment (n=3). CRT-NP + FUS (CFUS) resulted in the highest CRT expression and CRT to CD47 ratio. (H-I) CFUS enhanced tumor regression compared to CRT-NPs. Mice vaccinated s.c. in the flank with 4x10^6^ B16F10 cells transfected with CRT-NPs ± FUS (n=5) showed relatively slower tumor growth in CFUS cohorts than CRT-NP. Data are shown as mean ± SEM. Statistics were determined by ANOVA followed by Fisher's LSD without multiple comparisons correction. Differences between control and CRT-NP were analyzed using an unpaired t test. * p < 0.05, ** p < 0.01.

For CRT-NP synthesis, CRT plasmids were encapsulated in the cationic liposomes composed of DOTAP and cholesterol (10: 1; lipid: plasmid; wt.: wt). Compared to the free CRT plasmid, agarose gel electrophoresis showed that DNA migration was absent for the CRT-NPs (**Figure 1A**), suggesting efficient plasmid encapsulation. The encapsulation was also evident in TEM where the CRT-NPs demonstrated a typical spherical core-shell morphology encapsulating the plasmid with an average size of ~230 nm **(Figure 1B)**. Additional characterizations by DLS in physiological buffer showed a hydrodynamic diameter of ~250 nm, zeta-potential of ~ +14 mv and a PDI <0.3 for the CRT-NP, and excellent stability in physiological buffers up to several days. NPs with a positive charge are efficiently taken up by cells 27. To determine whether this was true in case of CRT-NPs, fluorescence imaging and flow cytometry analysis of B16F10 melanoma cells incubated with CRT-NPs were performed. A significantly enhanced uptake of coumarin-labeled CRT-NPs at 5 h relative to untreated control was noted with flow cytometry. Also, the MFI signals plateaued at ~ 8 h, and started to decrease at 24h, indicating NP lysis (**Figure 1C**). To assess whether the enhanced NP uptake translated into an increased CRT expression in the B16F10 cells relative to the un-transfected control, fluorescence imaging of the treated cells were performed at 15, 24 and 48 h post transfection**.** Compared to control, plasmid, and blank NP, our data suggested a significant and progressive increase in CRT expressions over 48h similar to the LF2000 positive control **(Figure 1D)**. These were also verified in quantitative flow assays where the CRT expression was found to be ~2-fold higher for the CRT-NPs compared to un-transfected control (**Figure 1E**). Next, we assessed the role of FUS in CRT-NP therapy. Adding FUS to CRT-NPs (CFUS) can hypothetically enhance membrane translocation of CRT, and modulate the CRT/CD47 axis by thermal effect. To assess this mechanism***,***B16F10 cells transfected with CRT-NPs were exposed to FUS heating *in vitro* (42-45°C). Data suggested that CRT-NP+FUS (CFUS) was most effective in inducing CRT expressions (3-fold higher) compared to the untreated control **(Figure 1F).** Also, in contrast to the CRT-NPs for which the enhanced expression of CRT was accompanied by a concurrent upregulation of CD47, the CFUS treatment increased the CRT without significantly changing the CD47 membrane expression, thereby resulting in a 1.5-fold increase in CRT/CD47 ratios compared to the control, FUS and CRT-NP **(Figure 1G).** Finally, to confirm whether downregulating the CD47 expression induced tumor regressions in vivo, we inoculated the mice (n=5) subcutaneously with CRT-NP and CFUS treated cells in the flank regions **(Figure 1H)**. A significantly superior tumor regression for CFUS relative to CRT-NP treated cells (n=5) was noted in the mice over 4-week, suggesting that CFUS directly prevented the CD47 counteraction of CRT expression in melanoma cells to improve the therapeutic response **(Figure 1I).**

### Local CRT-NP and CFUS tumor therapy enhanced ICD and therapeutic efficacy in vivo

**Figure 2:**
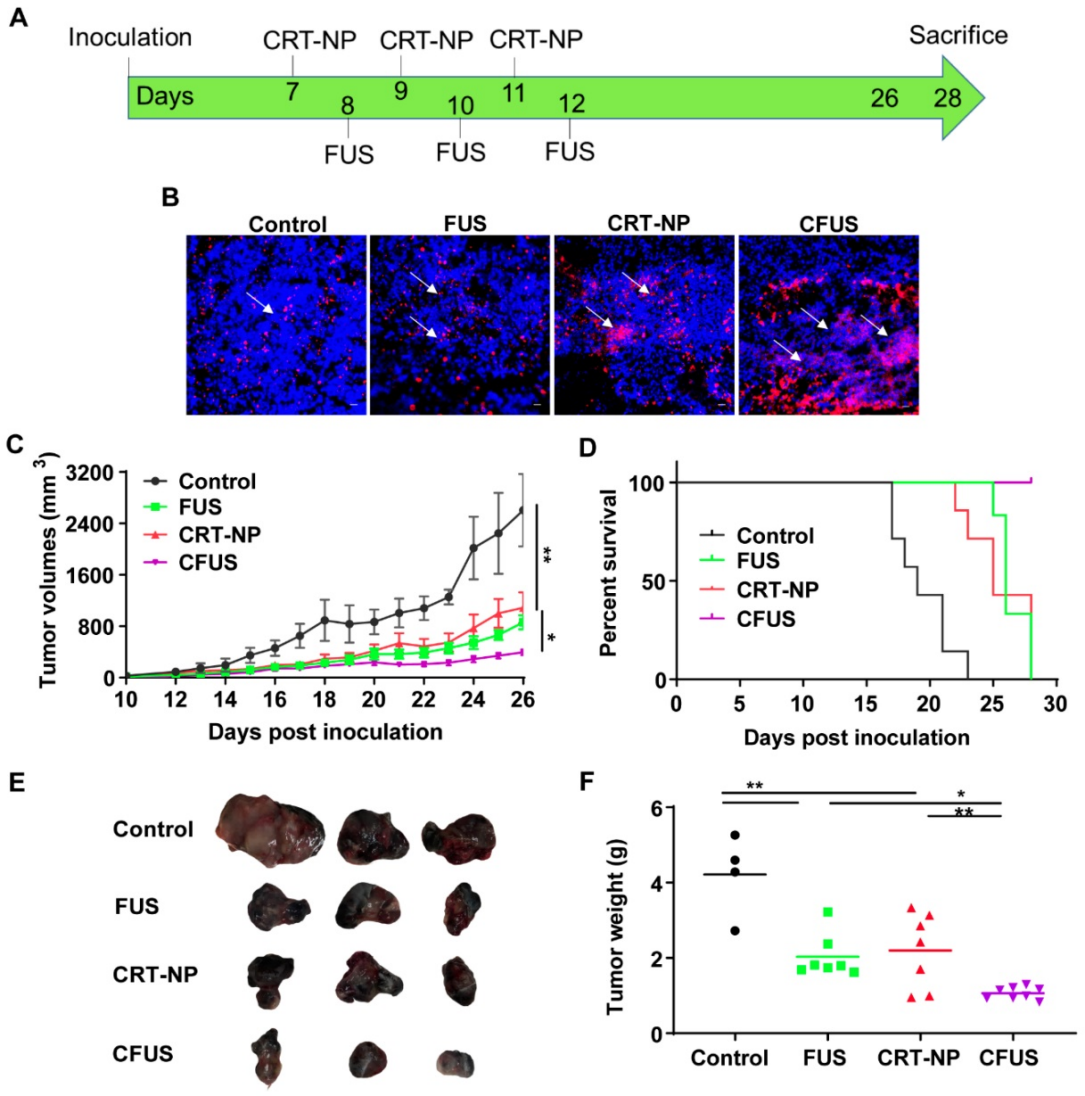
CRT-NP and FUS local treatment enhanced therapeutic efficacy in vivo and synergized when combined as CFUS. (A) Experimental design to test the efficacy of CFUS against melanoma tumors. (B) Immunofluorescence images of B16F10 melanoma tumor sections showing CRT expression (red) and nuclei (DAPI blue). CFUS significantly enhanced CRT intensity in treated tumors compared to other groups (10X magnification). (C) Growth curves of mice tumors in various experimental groups. CFUS significantly achieved tumor growth delay compared to Control, FUS, and CRT-NPs (n = 4-7). (D) Differences in the survival were determined for each group by the Kaplan-Meier method and the overall P value was calculated by the log-rank test. (E) Representative images of the harvested tumor. (F) Tumor weights at the time of sacrifice showed significant reduction in the overall weight with treatments compared to control. Statistics were determined by ANOVA followed by Fisher's LSD without multiple comparisons correction. * p < 0.05, ** p < 0.01.

We evaluated the CRT-expression and efficacy of CRT-NP and FUS heating by tumor growth and weight measurements in B16F10 melanoma model over 26-28 days **(Figure 2A).** When FUS was combined with CRT-NP, the CRT expression in tumors were significantly upregulated in the fluorescence imaging **(Figure 2B).** Therapeutically, the untreated control mice exhibited pronounced increases in tumor volume **(Figure 2C).** CRT-NP alone and FUS treatments induced significant tumor volume reductions (~50%) compared to the control but did not differ significantly from each other. In contrast, the CFUS treatments caused significant suppression of tumor growth rates (> 85%) vs. that seen in untreated controls and an effect that was ~50% greater than that seen with CRT-NP or FUS alone over the period of treatment. Evaluation of survival rates indicated that the CFUS treated mice (n=7) demonstrated 100% survival vs 70% for CRT-NP (2/7). In contrast, FUS (0/7), and control (0/7) were ineffective in tumor control and survival compared to CRT-NP and CFUS **(Figure 2D)**. Furthermore, CFUS significantly decreased the tumor weight to a greater extent by visual and statistical measures compared to all other groups **(Figure 2E*-*F).**

### CRT-NP and CFUS induced ICD increased the infiltration of tumor suppressing immune cell

**Figure 3:**
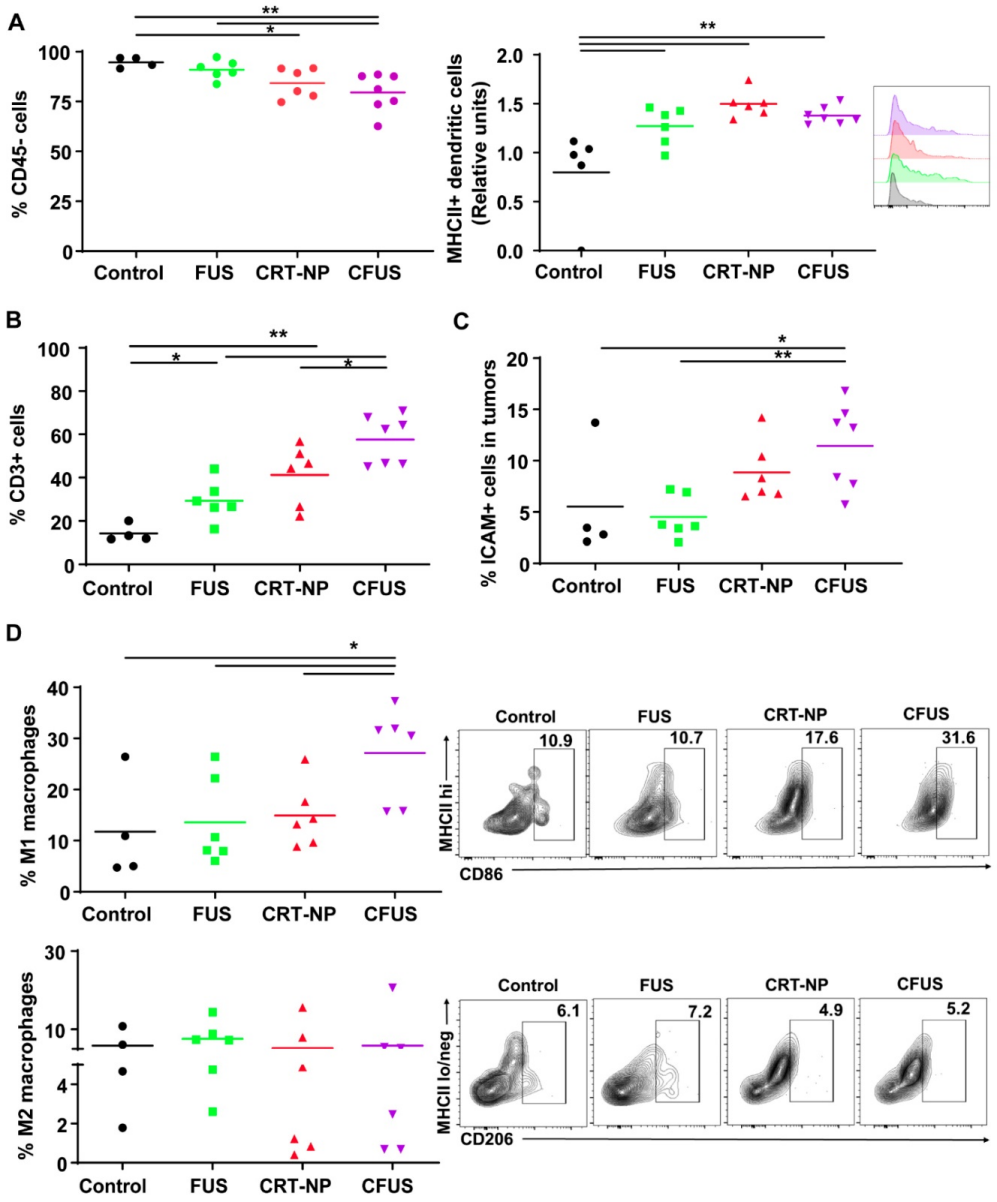
Local CRT-NP and FUS therapy activated antigen presenting cells and induced infiltration of T-cells in the tumor. (A) Percentage of CD45 minus (-) cells (tumor cells and fibroblasts) was decreased with CFUS and CRT-NP therapy compared to untreated control. Infiltration of dendritic cells expressing activation marker namely MHCII was enhanced by monotherapies and CFUS. Histograms plots showed an increase in median fluorescence intensity (MFI) of MHCII expressed on intratumoral dendritic cells for the treatments compared to control. (B) Population of CD3+ cells increased by 2-3-fold for FUS, CRT-NP and CFUS compared to control. (C) CFUS tumors showed ~2-fold higher ICAM-1 expression than control. (D) Percentage of macrophages (CD11b+ F4/80+) in the tumors were analyzed to determine M1 and M2 subtypes. MHCIIhi CD86+ for M1 showed significant increase with CFUS and MHCIIlo/neg CD206+ was unaltered for the M2 subtypes in treated tumors. Data are shown as mean ± SEM, one-way ANOVA followed by Fisher's LSD without multiple comparisons correction. Differences between control and treatments in C were analyzed using an unpaired t test assuming unequal variance. * p < 0.05, ** p < 0.01.

We determined within B16F10 melanoma tumors the percentage of the non-immune (tumor and fibroblast cells) and dendritic cell (DC), tumor associated macrophage (TAM) phenotypes, and T cells. The percentage of CD45 minus cells (tumor cells and fibroblasts; mean ± SEM) in tumors were 95 ± 1.32, 90.9 ± 1.91, 84.2 ± 3.1, and 79.5 ± 3.6 for control, FUS, CRT-NP and CFUS, respectively. Also, the MHCII expression on DC (CD11b+ CD11c+ F4/80-) was relatively higher in the treated tumors compared to untreated control, indicating activation of immune system **(Figure 3A*)*.** The decrease in tumor cell population for CFUS and CRT-NP strongly correlated with the infiltration of CD3+ T cells **(**2-3-fold**, Figure 3B),** and overall ICAM-1 levels in tumors **(2-fold, Figure 3C).** In addition, CFUS significantly enhanced TAMs (CD11b+ F4/80+) of the M1 phenotype (MHCIIhigh; 3-fold) without impacting the M2 population (MHCIIlow/negative CD206+; **Figure 3D).**

### CRT based ICD improved the local and systemic anti-tumor immunity compared to untreated control

**Figure 4:**
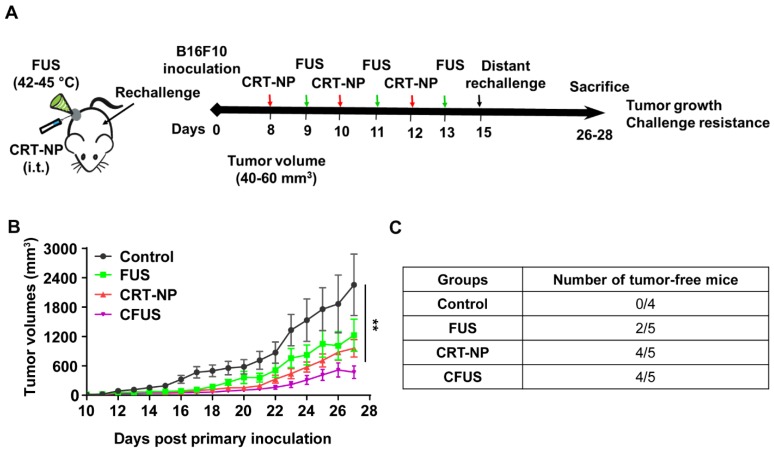
CFUS improved the local and systemic anti-tumor immunity in B16F10 melanoma model. (A) Mice were challenged in the contralateral flank with 1x10^5^ B16F10 cells 2 weeks post inoculation of the primary tumor (n=4-5). (B) Tumor volumes at the treated tumor site showing significant regression for CFUS and CRT-NPs despite pressure imposed on the immune system by tumor re-challenge. (C) Number of mice that were tumor free at the distant untreated site. Data are shown as mean ± SEM, * p < 0.05, ** p < 0.01; One-way ANOVA followed by Fisher's LSD without multiple comparisons correction.

To determine the role of CRT induced ICD in inducing resistance against re-challenge, mice (n=4-5) were randomized as follows: control, FUS, CRT-NPs, and CFUS (**Figure 4A**). Briefly, the primary tumors were treated at a volume of 40-60 mm^3^, and then mice were re-challenged with 1x10^5^ B16F10 cells s.c. on the contralateral flank on day 7 and observed for 14 days for tumor growth. Data suggested that the sustained pressure on the immune system by tumor re-challenge did not impact therapeutic effects at the primary treated site. Overall, CFUS was most effective in local control relative to untreated control, FUS, and CRT-NP during the longitudinal monitoring period **(Figure 4B).** In addition, 80% of tumor-bearing mice (4/5) in CFUS and CRT-NP remained tumor-free and resisted challenge at distal site compared to 40% mice (2/5) in FUS and 0% (0/4) in the untreated control **(Figure 4C).**

### ICD mediated by CRT-NP and CFUS induce melanoma specific immunity

**Figure 5:**
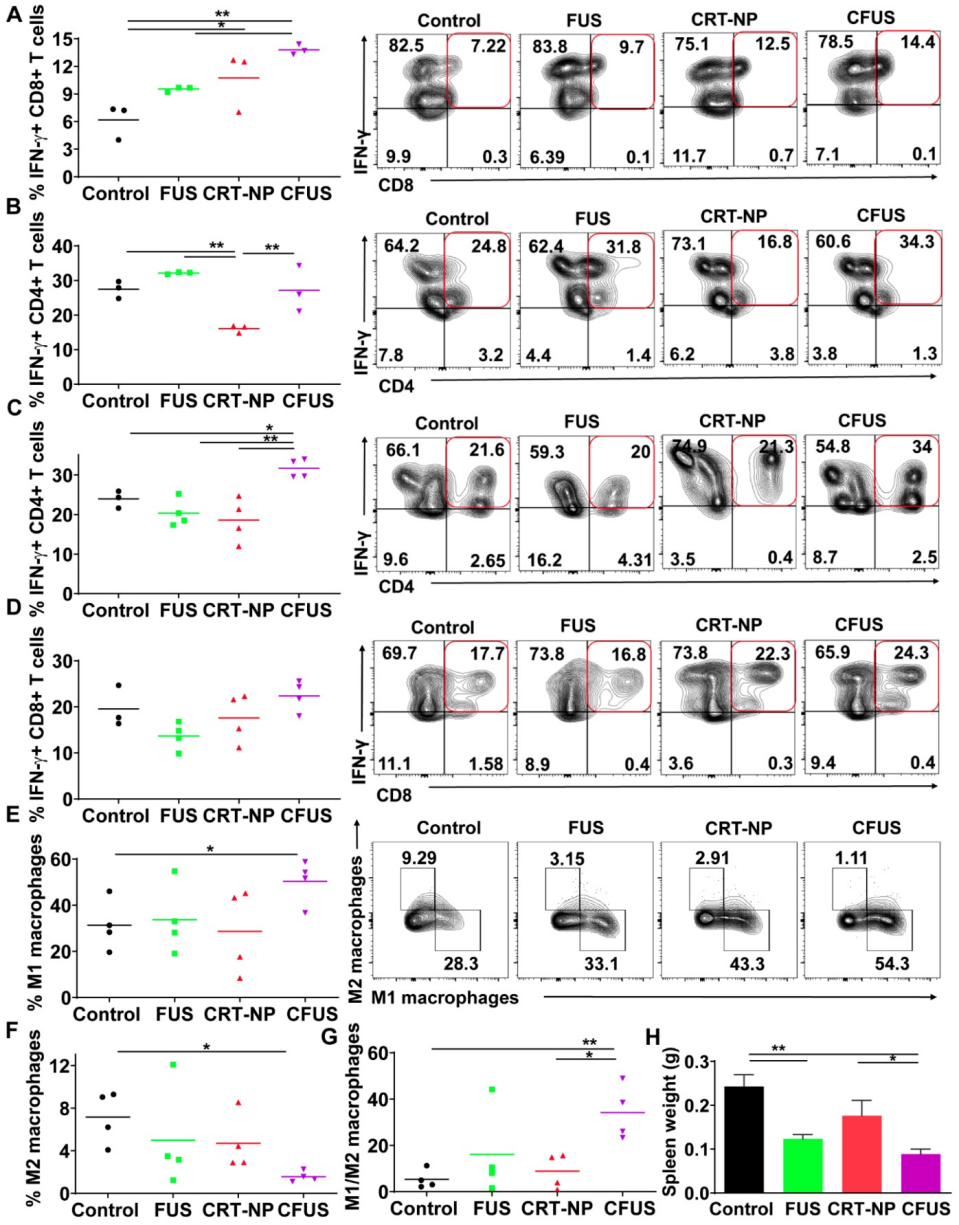
Evaluation of melanoma specific local and systemic immunity in the draining lymph nodes (dLN) and splenic tissue from the mice sacrificed on the same day post inoculation. (A and B) IFN-γ secreting CD8+ T cells in the dLN after ex-vivo stimulation with TRP-2 melanoma antigen showed 1.5-3-fold increase for CRT-NP, FUS and CFUS, compared to untreated control (n=3). IFN-γ+ CD4+ T cells did not change between the treatments compared to control. (C and D) IFN-γ producing CD4+ T cells in the spleen after TRP-2 stimulation were enhanced by CFUS compared to the other groups. IFN-γ+ CD8+ T cells were not altered in the treatment groups (n=3-4). (E) Frequency of M1 macrophages in the spleen was increased by ~2-fold for CFUS compared to FUS, CRT-NP and control. (F-G) M2 macrophages decreased with CFUS therapy resulting in higher M1 to M2 ratio in the spleen than other cohorts. (H) Weights of the spleen from mice were significantly lowered for the various treatment groups compared to control. Data are shown as mean ± SEM. Statistics were determined by ANOVA followed by Fisher's LSD without multiple comparisons correction. Differences between control and treatments in E were analyzed using an unpaired t test assuming unequal variance * p < 0.05, ** p < 0.01.

**Figure 6:**
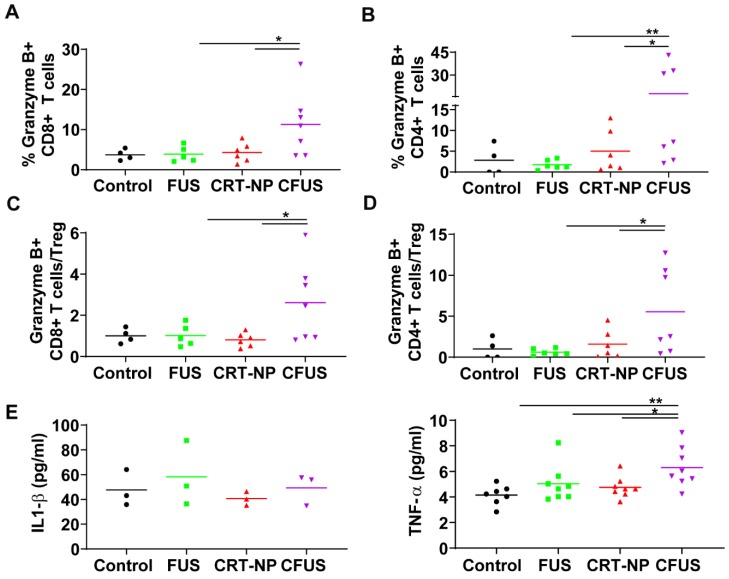
CFUS enhanced granzyme-B expression on tumor infiltrating T cells. (A and B) CFUS treated tumors showed a higher percentage of granzyme B+ CD8 and CD4 T cell population than other groups (n=4-7). (C and D) Calculated ratios of % granzyme B+ T cells to Foxp3+ CD4+ Tregs (MFI) were the highest for CFUS tumors (n=4-7). (E) Intratumoral cytokines measured by ELISA. IL-1β was not altered in the treated tumors (n=3). CFUS treatment resulted in a significant increase in TNF-α compared to FUS or CRT-NP alone (n=7-8). Data are shown as mean ± SEM, * p < 0.05, ** p < 0.01; One-way ANOVA followed by Fisher's LSD without multiple comparisons correction.

To determine melanoma antigen-specific immune response in the mice model, we assessed the production of IFN-γ from T cells in the dLNs and splenic tissue of surviving mice. An increase in IFN-γ+ CD8+ T cells (~2-fold fold) in dLNs in CRT-NP, CFUS, and FUS were observed, but not IFN-γ+ CD4+ T cells indicating the proliferation of melanoma specific cytotoxic immune cells (**Figure 5A-B**). In contrast, the splenocytes from various groups that were cultured ex vivo and stimulated with TRP-2 melanocyte antigen showed a significant enhancement of IFN-γ+ CD4+ T cells for CFUS compared to CRT-NP. However, the IFN-γ+ CD8+ T cells were not altered significantly between groups **(Figure 5C-D).** We also analyzed the splenic macrophages for the M1/M2 phenotype and tumor T cell phenotypes to understand why CFUS induced the most prominent antitumor effects following local treatments compared to all other groups. Results showed a 1.5-2-fold increase in M1 subsets (**Figure 5E**) and 4-5-fold increase in M1/M2 ratio for CFUS (34.2 ± 5.1) compared to control (5.5 ± 2.4), FUS (16.1 ± 8.3), and CRT-NP (8.9 ± 3.2; **Figure 5F-G**). These changes in the M1/M2 ratio accompanied a significant decrease in the splenic weight for treatment cohort's vs control (**Figure 5H**). Importantly, for the CFUS group, the population of functional T cells demonstrated the highest population of CD3+CD8+ and CD4+T cells expressing g*ranzyme* B, a key activation marker involved in tumor cell lysis 39 (**Figure 6A and B**; % Granzyme B+ CD8+ T cells; CFUS: 11.29 ± 3.0, Control: 3.72 ± 0.68, FUS: 3.88 ± 3.40, and CRT-NPs: 4.31 ± 0.97 and for % Granzyme B+ CD4+ T cells; CFUS: 17.85 ± 6.46, Control: 2.82 ± 1.78, FUS: 1.77 ± 0.46, and CRT-NPs 5.01 ± 2.12). This increase in the activated T cells correlated with increased ratios of CD8+ and CD4+ T cells to Tregs for CFUS compared to other groups **(Figure 6C and D).** Lastly, the immuno-activated profile in tumors accompanied an increase in the expressions of TNF-α in CFUS tumors. In contrast, the expression of IL-1β was not altered between groups (**Figure 6E**).

### Adaptive resistance can emerge in melanoma tumors following withdrawal of CRT-ICD therapy

**Figure 7:**
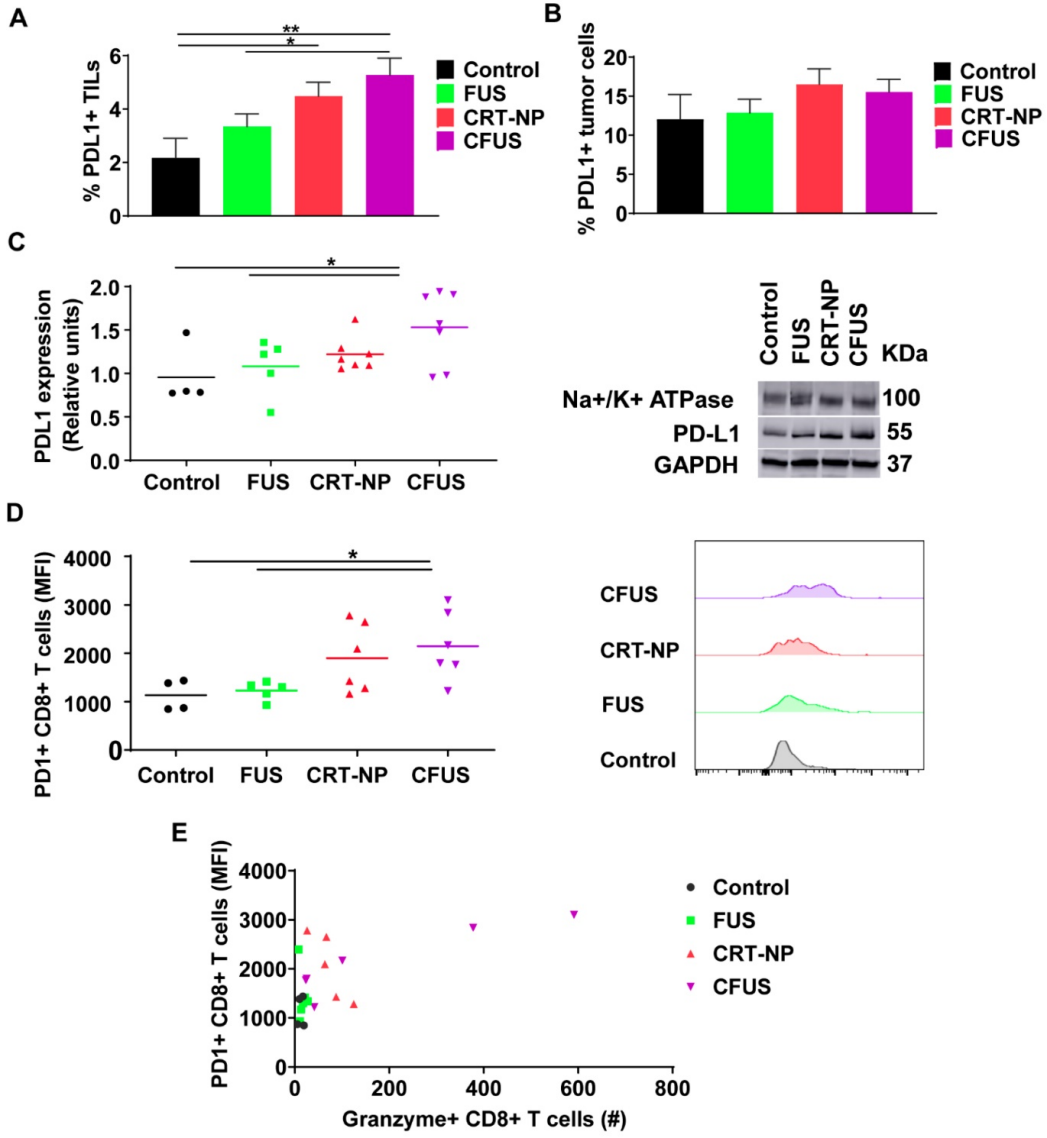
CRT-based ICD therapy modulated the checkpoint markers in tumors and T cells. The surface expression of PD-L1 and PD-1 in treated tumors was analyzed using flow cytometry and western blot and shown as mean ± SEM. (A) Percentage of PD-L1+ TILs were significantly enhanced by CFUS and CRT-NP (n=3-4). (B) Frequency of PD-L1+ tumor cells did not change with treatments. (C) Representative western blots of PD-L1 from crude membrane fractions of tumors. Na+/K+ ATPase was used as a loading control (n=4-7). (D) PD-1 expression on CD3+ CD8+ T cells represented as median fluorescence intensity (MFI). Histogram plots indicate the difference in PD-1 MFI among groups. (E) The relationship between the PD-1+ CD8+ T cells and Granzyme B+ CD8+ T cells showed correlation in responding mice. Pearson's r = 0.644, p < 0.01. Statistics were determined by ANOVA followed by Fisher's LSD without multiple comparisons correction. * p < 0.05, ** p < 0.01.

To understand the role of ICD therapy in PD-L1/PD-1 pathway on tumor cells and TILs, we assessed expression of PD-L1 and PD-1 in tumors. Flow cytometry and western blot data suggested that the PD-L1 expressions on tumor cells were not modified by the treatments, but the frequency of PD-L1+ TILs from CFUS tumors was significantly increased (~2.5 folds) compared to control **(Figure 7A-C).** Additionally, CFUS treatment enhanced the median fluorescence intensity (MFI) of PD-1 on CD3+ CD8+ T cells compared to CRT-NP, control and FUS **(Figure 7D).** This enhancement was particularly associated with the Granzyme B+ CD8+ T cells (r=0.644, p < 0.01, **Figure 7E**). Notably, the mice that expressed highest levels of Granzyme B+ and PD-1 CD8+ T cells demonstrated superior tumor regression (especially in CFUS group). In contrast, low levels of granzyme B and PD-1 expression resulted in sub-optimal therapeutic outcomes.

## Discussion

The goal of this study was to understand how CRT based ICD impacts the infiltration of immune cell, antigen cross-presentation, and antitumor immunity in murine melanoma. CRT is a pro-phagocytic signaling protein that translocates to tumor cell membranes during cellular stress to promote immunogenicity of tumors 14. CRT expression is mainly mediated by the chemo-, radio-, and ablative therapy, but the extent of expression rates, antigen recognition and immune cell trafficking can be highly variable 15, 19, 23, 40, 41. To overcome this barrier, and achieve robust ICD, we developed liposome-based CRT-NP. In vitro data suggested that the synthesized NPs successfully encapsulated the plasmid (Figure 1A & 1B) and achieved high uptake and transfection efficiencies and surface exposure of CRT in melanoma cells in vitro and in vivo (Figure 1C-E, and Figure 2B). In contrast to CRT, CD47 (integrin-associated protein, IAP) acts as a negative checkpoint (don't eat me) of the innate immune system by interacting with signal-regulatory protein α (SIRPα) on macrophages, preventing their phagocytosis 42, 43. Because CD47 counterbalances the CRT pro-phagocytic and adaptive immunity, it may impact maximum synergy and durable responses from CFUS therapies. Our in vitro and in vivo studies suggest that FUS directly prevented the CD47 counteraction of CRT expression in melanoma cells, significantly improving therapeutic regression of tumors (Figure 1F-H). Additional future studies that include anti-CD47 antibody in the treatment regimen can shed more insights on this important phenomenon to optimize clinical outcomes, especially in scenarios where a proportional increase in the don't-eat-me signals, such as CD47 with CRT are noted.

An enhanced surface translocation of CRT followed by ICD is known to activate innate and adaptive immune cells 12, 14, 38. To test whether this was true in our model system, we determined the infiltration of M1 macrophages in the treated tumor and spleen upon in-situ vaccination with CRT-NP. CRT-NP and the addition of FUS in the CRT-NP regimen achieved a 2-fold increase in M1/M2 ratio compared to control and CRT-NP treatments (Figure 3D, and Figure 5E-G), verifying prior published findings wherein an increased M1/M2 ratio was associated with improved patient survival 44, 45. This phenomenon is typically attributed to the ability of M1 macrophages to secrete complement factors that facilitate phagocytosis, present antigens to T cells, and effectively shape an adaptive immune response 46. In contrast, high populations of M2-macrophages can promote tumor initiation, progression, and metastasis 47. Recent works in murine mammary, colon and melanoma cancers have also shown the presence of non-M1/M2 macrophage subtypes rich in IFN-γ section, T-cell receptor and CD169 expressions, and receptors with collagenous structure (MARCO) with M2-like profile 48-50. While our data seems to suggest that M1 phenotype was enhanced, more detailed studies may be required to correctly delineate the macrophage sub-populations that are involved in antitumoral effects with ICD. Like macrophages, dendritic cells play an important role in initiating an adaptive immune response by processing tumor antigens and presenting peptide fragments to activate naive CD4+ and CD8+ T cells, aiding in the clonal expansion of cytotoxic T lymphocytic cells, and improved therapeutic outcomes 51-55. We found that the treated mice tumors demonstrated a higher frequency of T cell and DCs following ICD with CRT-NP and CFUS (Figure 3A-B). Surprisingly, we did not observe significant alterations in intratumoral IL-1β levels, a pro-inflammatory cytokine produced from activated macrophages that is involved in T cell activation 56, 57. This said, our phenotypic characterization of CD4+ and CD8+ T cells in tumors from the treated mice revealed up-regulation of granzyme B along with an intratumoral increase in TNF-α especially for the CFUS group compared to monotherapies (Figure 6A-E), and this correlated strongly with the antitumor effects (Figure 2 and 4). IFN-γ, TNF-α, and granzyme B are typically associated with antitumor activity of cytotoxic CD8+ T cells via induction of enhanced tumor cell arrest and apoptosis 10, 58-60. Together, our data suggest that ICD induces tumor inflammation via multiple interrelated pathways, leading to tumor regression. This is highly promising, but the CRT translocation rates, cytokine expressions, and the immune activation can also vary depending on FUS acoustic parameters. Studies are currently underway in our laboratory to delineate the role of FUS parameter on the immune infiltrations, and their relationships with CD47, granzyme B and cytokine expression profile for more durable outcomes.

A key challenge in immunotherapy regimens is the generation of tumor-specific T cells against distant untreated tumors 61. We found that transfection of B16F10 cells with CRT-NP and combination with FUS heating significantly enhanced the populations of IFN-γ+ CD4+ and CD8+ T cells (~1.5-2) in dLN and splenic tissues. IFN-γ producing T-cells promote the priming and expansion of cytotoxic cells 62, 63. We propose that CRT-ICD delays tumor growth in distant untreated site by increasing the tumor antigen-specific T-cell quantity and quality (Figure 5A-D). Finally, we also assessed the role of immune checkpoints such as PD-L1/PD-1 that negatively influences innate and adaptive immune system 64. When antigen-specific T cells surround the tumor cells, the tumor cells along the T-cell rich margin upregulate PD-L1 as an immune evasive mechanism. This compensatory elevation in PD-L1 expression is thought to be due to presence of activated T-cells and chronic IFN-signaling, leading to impaired tumor cell killing 32, 33, 65, 66. We observed that our treatments upregulated PD-1/PD-L1 protein on tumor infiltrating lymphocytes (TILs) compared to untreated control (Figure 7A and 7B). Additionally, the CD8+ T cells showed increased granzyme B and IFN- γ expression upon CFUS therapy (Figure 6C). Thus, we hypothesize that the ICD induced T cell activation and the concurrent presence of chronic IFN-γ secretion can contribute to PD-L1 expression and development of an adaptive immune resistance mechanism, and this may influence the overall therapeutic outcomes. To overcome this barrier, the inclusion of checkpoint inhibitors in the ICD regimens can likely result in significantly improved outcomes in such cases 32, 33, 65, 66. This is supported by our data where the tumors that showed superior regression with ICD (especially CFUS) contained higher populations of PD-1+/PD-L1+ CD8+ T cells and activated CD8+ T cells (granzyme expressing; Figure 7D). Unlike tumor cells, PD-L1 expression on TILs has been associated with favorable prognosis in head and neck cancer and melanoma 67-69. Likewise, high PD-L1+ expressing metastatic melanoma achieved a superior clinical response to check point blockades compared to PD-L1- metastatic melanoma 68. Thus, we propose that the presence of activated T-cells and PD-L1+ TIL cells, and inclusion of checkpoint blockade can mitigate adaptive resistance effects to some extent, and this mechanism needs to be probed in future.

In summary, our in vitro and in vivo data suggest that CRT-based ICD promote antigen presentation and infiltration of activated CD8+ T cells in tumors. Adding FUS to CRT-NP therapy modulate the CRT-CD47-PD-L1 axis, improving the overall local and systemic therapeutic effect in melanoma. Additional assessment of ICD synergism with checkpoint blockades, anti-CD40 antibodies, and different FUS parameters can provide more insights on mitigating adaptive immune resistance, maximizing therapeutic effect and survival.

## Abbreviations

CRT: Calreticulin
ICD: Immmunogenic Cell Death
NP: Nanoparticle
FUS: Focused Ultrasound
CFUS: CRT + FUS

## References

[1] Leach DR, Krummel MF, Allison JP (1996). Enhancement of antitumor immunity by CTLA-4 blockade. Science (New York, NY).

[2] Butte MJ, Keir ME, Phamduy TB, Sharpe AH, Freeman GJ (2007). PD-L1 interacts specifically with B7-1 to regulate T cell function (88.24).

[3] Curran MA, Montalvo W, Yagita H, Allison JP (2010). PD-1 and CTLA-4 combination blockade expands infiltrating T cells and reduces regulatory T and myeloid cells within B16 melanoma tumors. Proceedings of the National Academy of Sciences of the United States of America.

[4] Iwai Y, Ishida M, Tanaka Y, Okazaki T, Honjo T, Minato N (2002). Involvement of PD-L1 on tumor cells in the escape from host immune system and tumor immunotherapy by PD-L1 blockade. Proceedings of the National Academy of Sciences of the United States of America.

[5] Gorbet MJ, Ranjan A (2019). Cancer immunotherapy with immunoadjuvants, nanoparticles, and checkpoint inhibitors: Recent progress and challenges in treatment and tracking response to immunotherapy.

[6] Abe BT, Shin DS, Mocholi E, Macian F (2012). NFAT1 supports tumor-induced anergy of CD4(+) T cells. Cancer research.

[7] Rabinovich GA, Gabrilovich D, Sotomayor EM (2007). Immunosuppressive strategies that are mediated by tumor cells. Annu Rev Immunol.

[8] Zamarin D, Ricca JM, Sadekova S, Oseledchyk A, Yu Y, Blumenschein WM (2018). PD-L1 in tumor microenvironment mediates resistance to oncolytic immunotherapy. The Journal of clinical investigation.

[9] Wei SC, Anang N-AAS, Sharma R, Andrews MC, Reuben A, Levine JH (2019). Combination anti-CTLA-4 plus anti-PD-1 checkpoint blockade utilizes cellular mechanisms partially distinct from monotherapies. Proceedings of the National Academy of Sciences.

[10] Zamarin D, Holmgaard RB, Subudhi SK, Park JS, Mansour M, Palese P (2014). Localized oncolytic virotherapy overcomes systemic tumor resistance to immune checkpoint blockade immunotherapy. Science translational medicine.

[11] Obeid M (2008). ERP57 membrane translocation dictates the immunogenicity of tumor cell death by controlling the membrane translocation of calreticulin. Journal of immunology (Baltimore, Md: 1950).

[12] Obeid M, Tesniere A, Ghiringhelli F, Fimia GM, Apetoh L, Perfettini JL (2007). Calreticulin exposure dictates the immunogenicity of cancer cell death. Nature medicine.

[13] Panaretakis T, Joza N, Modjtahedi N, Tesniere A, Vitale I, Durchschlag M (2008). The co-translocation of ERp57 and calreticulin determines the immunogenicity of cell death. Cell death and differentiation.

[14] Wang HT, Lee HI, Guo JH, Chen SH, Liao ZK, Huang KW (2012). Calreticulin promotes tumor lymphocyte infiltration and enhances the antitumor effects of immunotherapy by up-regulating the endothelial expression of adhesion molecules. International journal of cancer.

[15] Galluzzi L, Buque A, Kepp O, Zitvogel L, Kroemer G (2017). Immunogenic cell death in cancer and infectious disease. Nat Rev Immunol.

[16] Green DR, Ferguson T, Zitvogel L, Kroemer G (2009). IMMUNOGENIC AND TOLEROGENIC CELL DEATH. Nature reviews Immunology.

[17] Apetoh L, Obeid M, Tesniere A, Ghiringhelli F, Fimia GM, Piacentini M (2007). Immunogenic chemotherapy: discovery of a critical protein through proteomic analyses of tumor cells. Cancer genomics & proteomics.

[18] Fucikova J, Becht E, Iribarren K, Goc J, Remark R, Damotte D (2016). Calreticulin Expression in Human Non-Small Cell Lung Cancers Correlates with Increased Accumulation of Antitumor Immune Cells and Favorable Prognosis. Cancer research.

[19] Garg AD, Dudek-Peric AM, Romano E, Agostinis P (2015). Immunogenic cell death. The International journal of developmental biology.

[20] Cui S (2017). Immunogenic Chemotherapy Sensitizes Renal Cancer to Immune Checkpoint Blockade Therapy in Preclinical Models. Med Sci Monit.

[21] Wang J, Gao ZP, Qin S, Liu CB, Zou LL (2017). Calreticulin is an effective immunologic adjuvant to tumor-associated antigens. Experimental and therapeutic medicine.

[22] Feng M, Chen JY, Weissman-Tsukamoto R, Volkmer J-P, Ho PY, McKenna KM (2015). Macrophages eat cancer cells using their own calreticulin as a guide: Roles of TLR and Btk. Proceedings of the National Academy of Sciences.

[23] Garg AD, Elsen S, Krysko DV, Vandenabeele P, de Witte P, Agostinis P (2015). Resistance to anticancer vaccination effect is controlled by a cancer cell-autonomous phenotype that disrupts immunogenic phagocytic removal. Oncotarget.

[24] Bing C, Nofiele J, Staruch R, Ladouceur-Wodzak M, Chatzinoff Y, Ranjan A (2015). Localised hyperthermia in rodent models using an MRI-compatible high-intensity focused ultrasound system. Int J Hyperthermia.

[25] Ektate K, Munteanu MC, Ashar H, Malayer J, Ranjan A (2018). Chemo-immunotherapy of colon cancer with focused ultrasound and Salmonella-laden temperature sensitive liposomes (thermobots). Sci Rep.

[26] Maples D, McLean K, Sahoo K, Newhardt R, Venkatesan P, Wood B (2015). Synthesis and characterisation of ultrasound imageable heat-sensitive liposomes for HIFU therapy. Int J Hyperthermia.

[27] Huang X, Yuan F, Liang M, Lo HW, Shinohara ML, Robertson C (2012). M-HIFU inhibits tumor growth, suppresses STAT3 activity and enhances tumor specific immunity in a transplant tumor model of prostate cancer. PloS one.

[28] Singh MP, Sethuraman SN, Ritchey J, Fiering S, Guha C, Malayer J (2019). In-situ vaccination using focused ultrasound heating and anti-CD-40 agonistic antibody enhances T-cell mediated local and abscopal effects in murine melanoma. Int J Hyperthermia.

[29] Bandyopadhyay S, Quinn TJ (2016). Low-Intensity Focused Ultrasound Induces Reversal of Tumor-Induced T Cell Tolerance and Prevents Immune Escape. J Immunol.

[30] Chao MP, Jaiswal S, Weissman-Tsukamoto R, Alizadeh AA, Gentles AJ, Volkmer J (2010). Calreticulin is the dominant pro-phagocytic signal on multiple human cancers and is counterbalanced by CD47. Science translational medicine.

[31] Liu X, Pu Y, Cron K, Deng L, Kline J, Frazier WA (2015). CD47 blockade triggers T cell-mediated destruction of immunogenic tumors. Nature medicine.

[32] Dosset M, Vargas TR, Lagrange A, Boidot R, Végran F, Roussey A (2018). PD-1/PD-L1 pathway: an adaptive immune resistance mechanism to immunogenic chemotherapy in colorectal cancer. Oncoimmunology.

[33] Benci JL, Xu B, Qiu Y, Wu T, Dada H, Victor CT-S (2016). Tumor Interferon Signaling Regulates a Multigenic Resistance Program to Immune Checkpoint Blockade. Cell.

[34] Thierry AR, Rabinovich P, Peng B, Mahan LC, Bryant JL, Gallo RC (1997). Characterization of liposome-mediated gene delivery: expression, stability and pharmacokinetics of plasmid DNA. Gene therapy.

[35] Hu Z, Yang XY, Liu Y, Morse MA, Lyerly HK, Clay TM (2005). Release of endogenous danger signals from HIFU-treated tumor cells and their stimulatory effects on APCs. Biochemical and biophysical research communications.

[36] Fan Y, Kuai R, Xu Y, Ochyl LJ, Irvine DJ (2017). Immunogenic Cell Death Amplified by Co-localized Adjuvant Delivery for Cancer Immunotherapy. Nano Lett.

[37] Toraya-Brown S, Sheen MR, Zhang P, Chen L, Baird JR, Demidenko E (2014). Local hyperthermia treatment of tumors induces CD8(+) T cell-mediated resistance against distal and secondary tumors. Nanomedicine: nanotechnology, biology, and medicine.

[38] De Palma R, Marigo I, Del Galdo F, De Santo C, Serafini P, Cingarlini S (2004). Therapeutic effectiveness of recombinant cancer vaccines is associated with a prevalent T-cell receptor alpha usage by melanoma-specific CD8+ T lymphocytes. Cancer research.

[39] Mellor-Heineke S, Villanueva J, Jordan MB, Marsh R, Zhang K, Bleesing JJ (2013). Elevated Granzyme B in Cytotoxic Lymphocytes is a Signature of Immune Activation in Hemophagocytic Lymphohistiocytosis. Frontiers in immunology.

[40] Dudek-Peric AM, Ferreira GB, Muchowicz A, Wouters J, Prada N, Martin S (2015). Antitumor immunity triggered by melphalan is potentiated by melanoma cell surface-associated calreticulin. Cancer research.

[41] Gameiro SR, Jammeh ML, Wattenberg MM, Tsang KY, Ferrone S, Hodge JW (2014). Radiation-induced immunogenic modulation of tumor enhances antigen processing and calreticulin exposure, resulting in enhanced T-cell killing. Oncotarget.

[42] Liu X, Kwon H, Li Z, Fu Y-x (2017). Is CD47 an innate immune checkpoint for tumor evasion?. Journal of Hematology & Oncology.

[43] Chao MP, Jaiswal S, Weissman-Tsukamoto R, Alizadeh AA, Gentles AJ, Volkmer J (2010). Calreticulin is the dominant pro-phagocytic signal on multiple human cancers and is counterbalanced by CD47. Science translational medicine.

[44] Jackute J, Zemaitis M, Pranys D, Sitkauskiene B, Miliauskas S, Sakalauskas R (2016). The prognostic influence of tumor infiltrating M1 and M2 phenotype macrophages in resected non-small cell lung cancer.

[45] Zhang M, He Y, Sun X, Li Q, Wang W, Zhao A (2014). A high M1/M2 ratio of tumor-associated macrophages is associated with extended survival in ovarian cancer patients. Journal of ovarian research.

[46] Sato-Kaneko F, Yao S, Ahmadi A, Zhang SS, Hosoya T, Kaneda MM (2017). Combination immunotherapy with TLR agonists and checkpoint inhibitors suppresses head and neck cancer.

[47] Jarosz-Biej M, Kamińska N, Matuszczak S, Cichoń T, Pamuła-Piłat J, Czapla J (2018). M1-like macrophages change tumor blood vessels and microenvironment in murine melanoma. PloS one.

[48] Georgoudaki AM, Prokopec KE, Boura VF, Hellqvist E, Sohn S, Ostling J (2016). Reprogramming Tumor-Associated Macrophages by Antibody Targeting Inhibits Cancer Progression and Metastasis. Cell reports.

[49] Chavez-Galan L, Olleros ML, Vesin D, Garcia I (2015). Much More than M1 and M2 Macrophages, There are also CD169(+) and TCR(+) Macrophages. Frontiers in immunology.

[50] Aras S, Zaidi MR (2017). TAMeless traitors: macrophages in cancer progression and metastasis. British journal of cancer.

[51] Kranz LM, Diken M, Haas H, Kreiter S, Loquai C, Reuter KC (2016). Systemic RNA delivery to dendritic cells exploits antiviral defence for cancer immunotherapy. Nature.

[52] Afreen S, Dermime S (2014). The immunoinhibitory B7-H1 molecule as a potential target in cancer: killing many birds with one stone. Hematology/oncology and stem cell therapy.

[53] Gooden MJ, de Bock GH, Leffers N, Daemen T, Nijman HW (2011). The prognostic influence of tumour-infiltrating lymphocytes in cancer: a systematic review with meta-analysis. British journal of cancer.

[54] Clark WH (1991). Tumour progression and the nature of cancer. British journal of cancer.

[55] Sato E, Olson SH, Ahn J, Bundy B, Nishikawa H, Qian F (2005). Intraepithelial CD8+ tumor-infiltrating lymphocytes and a high CD8+/regulatory T cell ratio are associated with favorable prognosis in ovarian cancer. Proceedings of the National Academy of Sciences of the United States of America.

[56] Haabeth OA, Lorvik KB, Yagita H, Bogen B, Corthay A (2016). Interleukin-1 is required for cancer eradication mediated by tumor-specific Th1 cells. Oncoimmunology.

[57] Luft T, Jefford M, Luetjens P, Hochrein H, Masterman K-A, Maliszewski C (2002). IL-1β Enhances CD40 Ligand-Mediated Cytokine Secretion by Human Dendritic Cells (DC): A Mechanism for T Cell-Independent DC Activation. The Journal of Immunology.

[58] Barth Jr RJ, Mule JJ, Spiess PJ, Rosenberg SA (1991). Interferon γ and tumor necrosis factor have a role in tumor regressions mediated by murine CD8+ tumor-infiltrating lymphocytes. Journal of Experimental Medicine.

[59] Benci JL, Liang YL, Nowell CJ, Halls ML, Wookey PJ, Dal Maso E (2016). Tumor interferon signaling regulates a multigenic resistance program to immune checkpoint blockade. Cell.

[60] Kearney CJ, Vervoort SJ, Hogg SJ, Ramsbottom KM, Freeman AJ, Lalaoui N (2018). Tumor immune evasion arises through loss of TNF sensitivity. Science Immunology.

[61] McWilliams JA, McGurran SM, Dow SW, Slansky JE, Kedl RM (2006). A modified tyrosinase-related protein 2 epitope generates high-affinity tumor-specific T cells but does not mediate therapeutic efficacy in an intradermal tumor model. Journal of immunology (Baltimore, Md: 1950).

[62] Deng W, Lira V, Hudson TE, Lemmens EE, Hanson WG, Flores R (2018). Recombinant <em>Listeria</em> promotes tumor rejection by CD8<sup>+</sup> T cell-dependent remodeling of the tumor microenvironment. Proceedings of the National Academy of Sciences.

[63] Bhat P, Leggatt G, Waterhouse N, Frazer IH (2017). Interferon-γ derived from cytotoxic lymphocytes directly enhances their motility and cytotoxicity. Cell Death &Amp; Disease.

[64] Liu B, Guo H, Xu J, Qin T, Guo Q, Gu N (2018). Elimination of tumor by CD47/PD-L1 dual-targeting fusion protein that engages innate and adaptive immune responses. mAbs.

[65] Minn AJ (2015). Interferons and the Immunogenic Effects of Cancer Therapy. Trends in Immunology.

[66] Spitzer MH, Carmi Y, Reticker-Flynn NE, Kwek SS, Madhireddy D, Martins MM (2017). Systemic Immunity is Required for Effective Cancer Immunotherapy. Cell.

[67] Kim HR, Ha S-J, Hong MH, Heo SJ, Koh YW, Choi EC (2016). PD-L1 expression on immune cells, but not on tumor cells, is a favorable prognostic factor for head and neck cancer patients. Scientific reports.

[68] Taube JM, Anders RA, Young GD, Xu H, Sharma R, McMiller TL (2012). Colocalization of Inflammatory Response with B7-H1 Expression in Human Melanocytic Lesions Supports an Adaptive Resistance Mechanism of Immune Escape. Science translational medicine.

[69] Zhao T, Li C, Wu Y, Li B, Zhang B (2017). Prognostic value of PD-L1 expression in tumor infiltrating immune cells in cancers: A meta-analysis. PloS one.

